# Lin28a up‐regulation is associated with the formation of restenosis via promoting proliferation and migration of vascular smooth muscle cells

**DOI:** 10.1111/jcmm.15506

**Published:** 2020-07-25

**Authors:** Zhiwei Zou, Xiaojun Zhou, Ruzhen Zhang, Qian Zhang, Shan Jiang, Chunmei Xu, Rui Zhang, Tianyue Xie, Huangao Zhu, Piyun Gong, Dongmei Zhang, Huimei Ma, Lin Liao, Jianjun Dong

**Affiliations:** ^1^ Department of Endocrinology Qilu Hospital Cheeloo College of Medicine Shandong University Jinan China; ^2^ Department of Endocrinology Yantai Affiliated Hospital of Binzhou Medical University Yantai China; ^3^ Department of Endocrinology and Metabology The First Affiliated Hospital of Shandong First Medical University Jinan China; ^4^ Department of Endocrinology and Metabology Shandong Qianfoshan Hospital Cheeloo College of Medicine Shandong University Jinan China; ^5^ Institute of Endocrine and Metabolic Diseases of Shandong University Jinan China; ^6^ Key Laboratory of Endocrine and Metabolic Diseases Shandong Province medicine and health Jinan China

**Keywords:** Lin28a, migration, percutaneous transluminal angioplasty, proliferation, restenosis, vascular smooth muscle cells

## Abstract

To explore the potential role of Lin28a in the development of restenosis after percutaneous transluminal angioplasty, double‐balloon injury surgery and mono‐balloon injury surgery were used to establish restenosis and atherosclerosis models, respectively, so as to better distinguish restenosis from atherosclerotic lesions. Immunohistochemical analysis revealed that significantly higher expression of Lin28a was observed in the iliac arteries of restenosis plaques than that of atherosclerosis plaques. Immunofluorescence studies showed the colocalization of Lin28a with α‐smooth muscle actin in restenosis plaques, rather than in atherosclerosis plaques, which suggested that Lin28a might be related to the unique behaviour of vascular smooth muscle cells (VSMCs) in restenosis. To further confirm above hypothesis, Lin28a expression was up‐regulated by transfection of Lenti‐Lin28a and inhibited by Lenti‐Lin28a‐shRNA transfection in cultured VSMCs, and then the proliferation and migration capability of VSMCs were detected by EdU and Transwell assays, respectively. Results showed that the proliferation and migration of VSMCs were significantly increased in accordance with the up‐regulation of Lin28a expression, while above behaviours of VSMCs were significantly suppressed after inhibiting the expression of Lin28a. In conclusion, the up‐regulation of Lin28a exerts its modulatory effect on VSMCs’ proliferation and migration, which may play a critical role in contributing to pathological formation of restenosis.

## 1 INTRODUCTION

The incidence of type 2 diabetes mellitus (T2DM) is rapidly soaring at an alarming rate, imposing a considerable burden on public health.^1^ Lower extremity atherosclerotic disease (LEAD), one of the frequent macrovascular complications of diabetes, accounts for one of the main factors causing foot ulceration and amputation, which leads to markedly reduced quality of life.^2^ LEAD commonly implicates the tibial/peroneal artery and/or their branches.^3^ Percutaneous transluminal angioplasty (PTA) is regarded as a primary approach in the treatment of LEAD.^4^, ^5^ However, restenosis which often occurs (incidence is 49.4% at 6 months after PTA and 52.5% at 1 year after PTA) in the dilated site of vessels after PTA restricts its long‐term efficacy.^6^ Treatment of LEAD is essential in preventing amputation, morbidity and mortality of diabetic patients.^7^ Thus, the prevention and therapy of restenosis have been the keen spot in the process of LEAD treatment.

Our previous study has demonstrated that restenosis and atherosclerosis had distinct pathogenesis mechanisms.^8^ Excessive migration and proliferation of vascular smooth muscle cells (VSMCs) were the dominant causes to develop the restenosis.^9^ However, the particular mechanism involved in it remains unclear. It is necessary to further carry on the research on the exact mechanism of restenosis occurrence and interdicting this process is of great effect on the prevention and therapy of restenosis.^10^ Therefore, we attempt to figure out the particular mechanisms of VSMCs’ migration and proliferation in restenosis based on the previous study.

Lin28 was originally identified in *Caenorhabditis elegans* (*C elegans*) as a component of the heterochronic gene pathway that regulates developmental timing.^11^ Lin28a, a homolog of Lin28, has been demonstrated to enhance tissue repair in some adult tissues in recent years.^12^, ^13^ It is reported that Lin28a reactivation can improve hair regrowth by promoting anagen in hair follicles and accelerate regrowth of cartilage, bone, and mesenchyme after ear and digit injury.^14^ The process of restenosis plaque formation by further impairment of atherosclerotic arteries by PTA resembles the above tissue repair,^15^ which speculated that Lin28a might be involved in the process of restenosis. However, the role of Lin28a in restenosis formation has not been elucidated yet.

In our research, restenosis and atherosclerosis rat models were established by using different methods, and the expression and the effect of Lin28a on the VSMCs’ proliferation and migration were further explored.

## 2 MATERIALS AND METHODS

### 2.1 Animals and treatments

Male Sprague‐Dawley (SD) rats (~120 g) were obtained from the Beijing Huafukang Bioscience Co. Inc. The rats were allowed to adapt to the new environment for at least 7 days, and then were fed with a high‐fat diet during the whole experiment. The animal care and experiments were in conformity to the previous publication.^8^ The protocol was authorized by the ethical committee of Qianfoshan Hospital Affiliated to Shandong University. All the rats were taken surgical procedures under anaesthesia with sodium pentobarbital.

High‐fat diet was provided for all rats in the whole experiment. Type 2 diabetes mellitus (T2DM) was induced by intraperitoneal injection of streptozocin (STZ, 27.5 mg/kg, Solarbio) to rats. After STZ injection for three days, the rat with fasting blood glucose (FBG) ≥11.1 mmol/L in three consecutive analyses was considered as a successful T2DM rat model. All eligible diabetic rats were assigned into three groups: restenosis, atherosclerosis and diabetic control group. Non‐diabetic rats were also included as the blank control group.

We developed models of restenosis and atherosclerosis in both diabetic rats. Surgical procedures for establishment of restenosis and atherosclerosis models were presented as followings.

### 2.2 Restenosis models

After diabetes model was successfully established for one week, rats were anaesthetized with phenobarbital. An arteriotomy in the right femoral artery of diabetic rats was conducted with standard surgical procedures.

Firstly, a 1.5‐mm, wire‐guided balloon catheter (Medtronic, Inc.) was inserted into the iliac artery through the femoral artery. The balloon was blown up at 8, 10 and 12 atmospheric pressure (atm), respectively. And then, the inflated balloon was hauled from iliac artery to the femoral artery to induce endothelial denudation. After the operation, the balloon catheter was withdrawn, the vessel was ligated, and the surgical site was closed. In order to induce restenotisis plaques, in the fourth weeks after the first surgical procedure, additional PTA was performed if the iliac artery developed severe atherosclerotic damage, which was confirmed by ultrasound examination (Visualsonics). After anaesthesia, a midsagittal incision was executed in the dissected distal ends of the right femoral artery. A 1.5‐mm, wire‐guided balloon catheter was reinserted into the narrow injured iliac artery and the balloon was inflated to 12 atm three times for 5 seconds each time at the stenosis site. After the dilatation, catheter was removed and the femoral artery was ligated.

### 2.3 Atherosclerosis models

Balloon‐injured endothelial denudation of the normal iliac arteries in diabetic rats was performed to develop atherosclerotic plaques as the rats in restenosis group underwent the first balloon dilatation on the same day.

### 2.4 EVG dye and Immunohistochemical staining

We chose the balloon‐induced injury part of iliac artery and selected the cross section of the diseased iliac artery for immunostaining and observation. Tissue paraffin sections were cut into pieces (5 and 4 µm for EVG and immunohistochemical staining, respectively), and were deparaffinized in xylene and rehydrated through graded alcohol. For EVG staining, the sections were placed in elastic stain solution for 30 minutes followed by differentiation with 5% ferric chloride solution. The slides were counterstained with Van Gieson solution for 10 seconds, and then were observed under microscopy. For immunohistochemical staining for α‐SMA and Lin28a, slides were incubated with the primary antibodies (rabbit anti‐α‐SMA, 1:400, Abcam; mouse anti‐Lin28a, 1:200, Santa Cruz Biotechnologies) overnight at 4°C. After rinsing by PBS, the sections were incubated with peroxidase enzyme‐conjugated goat anti‐rabbit secondary antibody (#PV9001, 1:400, Zhongshan) and peroxidase enzyme‐conjugated goat anti‐mouse secondary antibody (#PV9002, 1:400, Zhongshan) for 30 minutes at 37°C. Diaminobenzidine tetrahydrochloride (ZSBIO) in PBS was used to produce a brown colour. Then the sections were counterstained with haematoxylin.

### 2.5 Double immunofluorescence staining

Double immunofluorescence staining was performed to detect the colocalization of Lin28a and α‐SMA, a marker of VSMCs in tissue, and the colocalization of CD31 and α‐SMA in tissue, respectively. The tissue sections were incubated with mouse polyclonal anti‑Lin28a (1:200, Santa Cruz Biotechnologies) and rabbit anti‐α‐SMA (1:200, Abcam) antibody overnight at 4°C. Additionally, mouse polyclonal anti‑CD31 (1:200, Abcam) and rabbit anti‐α‐SMA (1:200, Abcam) antibody were used overnight at 4°C. After rinsing by PBS for three times, immunoreactivity products were visualized by incubation with appropriate Alexa Fluor 488‐conjugated secondary antibodies (1:50; Invitrogen), 594‐conjugated secondary antibodies (1:150; Invitrogen), along with DAPI (Solarbio) stain to visualize the nuclei. After rinsing, specimens were examined with a fluorescence microscope (OLYMPUS FSX100).

### 2.6 VSMCs isolation, purification and verification

Primary VSMCs were obtained by using explant culture method as described previously.^16^ In brief, 6‐week‐old SD rats (sex unlimited) were anaesthetized with sodium pentobarbital (50 mg/kg ip), and the iliac artery was quickly excised and placed in cold Dulbecco's modified Eagle's medium (DMEM, with 5 mmol/L glucose, Gibco, ThermoFisher). The medial VSMCs layers were cut into 1 mm^3^ tissues, and then were explanted in culture flask with DMEM medium containing 20% foetal bovine serum (FBS, Invitrogen, ThermoFisher), streptomycin (100 μg/mL) and penicillin (100 U/mL) and incubated in a humidified atmosphere with 5% CO_2_ at 37°C. The medium was changed twice a week, and the cells should be passaged when grown to 80% confluence.

We purified VSMCs according to differential adherence of various cells. After the cell mixture was isolated, we digested the cell mixture by 0.25% tyrisin. And then the cell mixture was washed by serum‐free M199 medium, and was resuspended by M199 complete medium. The culture flask containing cell mixture was standing for 40 to 50 minutes, and the fibroblast and endothelial cells were firstly adhered to the flask. Then, the culture flask was inverted, and the cell suspension was transferred into another culture flask. Repeating the above operation again and centrifuging the suspension. The cell pellet of VSMCs was retained after discarding the supernatant and resuspended into the culture flask.

In view of the specificity of SM22α expression in VSMCs, VSMCs were verified by immunofluorescence that all cells of SM22α and α‐SMA were positively stained, which proved that the extracted primary cells were indeed VSMCs.

### 2.7 Production and transfection of lentivirus vectors

Lentivirus carrying Lin28a shRNA (Lenti‐Lin28a‐shRNA) or Lin28a cDNA (Lenti‐Lin28a) was designed and produced by GeneChem Company. And VSMCs at the 3rd passage were transfected by above lentivirus. Lenti‐Lin28a, Lenti‐Lin28a‐shRNA and corresponding empty lentiviral vector (Lenti‐Lin28a‐shNC) were used to be transfected into VSMCs.

### 2.8 Proliferation and migration assays

The proliferation of VSMCs in vitro was determined using a 5‐ethynyl‐2′‐deoxeuridine (EdU) assay kit (RiboBio Co., Ltd). VSMCs were resuspended at 5 × 10^3^ cells/100 µL seeded in the 96‐well plate (Costar, Solarbio) after transfected by Lenti‐Lin28a, Lenti‐Lin28a‐shRNA and Lenti‐Lin28a‐shNC. Then, cells were incubated with 50 μmol/L of EdU for additional 2 hours at 37°C. After incubation, VSMCs were fixed with 4% paraformaldehyde and then incubated with 100 μL of 1 × Apollo reaction cocktail for 30 minutes. The DNA contents of VSMCs were stained with Hoechst 33342 for 10 minutes, and the EdU‐positive‐stained cells were counted under a fluorescence microscope (OLYMPUS FSX100).

Cell migration was evaluated by the Transwell chamber as described previously.^17^ After transfected by Lenti‐Lin28a, Lenti‐Lin28a‐shRNA and Lenti‐Lin28a‐shNC, VSMCs at 1.5 × 10^4^ cells/100 µL were seeded in the upper chambers of Matrigel‐coated 8‐μm pore size transwell filters (Corning Life Sciences). In the lower chamber, 500 μL DMEM containing 10% foetal bovine serum was added, which used as a chemoattractant. After incubation for 24 hours, the cells that migrated to the lower surface of the filter were fixed in 4% paraformaldehyde for 15 minutes, and stained with crystal violet, and the nonmigrating cells in the upper surface of the filter were removed with a cotton‐tipped swab. Then, the lower side of the filters was fixed in 4% paraformaldehyde for 15 minutes, and stained with crystal violet dyes. The images of migrated cells were taken at randomly selected fields using an OLYMPUS FSX100 imaging system (Olympus). The migration of VSMCs was evaluated as the mean number of cells in five randomly selected fields. The assay was repeated at least three times.

### 2.9 Quantitative real‐time polymerase chain reaction (qRT‐PCR)

RNA levels were detected by qRT‐PCR with One Step PrimeScriptmiRNA cDNA Synthesis Kit (Takara Bio Inc.).^18^ The following conditions were used: 95°C for 30 seconds, 95°C for 5 seconds and 60°C for 20 seconds for 40 cycles. The relative expression level of Lin28a mRNA was calculated using the following formula: relative gene expression = 2^−(ΔCtsample−ΔCtcontrol)^. The primer sequences were shown as following: Lin28a: Forward: 5’‐CAAGTGTCCTGCCCTGCTGTA‐3’, Reverse: 5’‐CCCAATGTGTTCTATTGCATTTGTC‐3’; β‐actin: Forward: 5’‐CATCCTGCGTCTGGACCT‐3’, Reverse: 5’‐GTACTTGCGCTCAGGAGGAG‐3’.

### 2.10 Western blot analysis

Protein concentrations in cell extracts were determined by Bio‑Rad DC Protein Assay kit (Bio‑Rad Laboratories, Inc.). Equal amounts of protein fractions of lysates were resolved over 10% SDS‐PAGE and transferred to PVDF membrane (EMD Millipore). The membranes were incubated with rabbit anti‐Lin28a (1:1000, Santa Cruz Biotechnologies), mouse anti‐β‐actin (1:1,000; Sigma‐Aldrich; Merck KGaA) overnight at 4°C. The next day, corresponding peroxidase‑labelled goat anti‑rabbit and anti‐mouse secondary antibody (1:5000) were used as the second antibody, respectively. The peroxidase activity was detected using the FluorChem E enhanced chemiluminescent system (ProteinSimple). And ImageJ software (National Institutes of Health) was used to quantify the densitometer of the optical density of the bands.

## 3 RESULTS

### 3.1 Animals

Total forty male SD rats were injected streptozotocin. Thirty of them developed hyperglycaemia, six died and four were excluded because of inadequate glucose levels. Thirty diabetic rats were randomly assigned to atherosclerosis and restenosis groups. After all surgical procedures, fifteen rats died during the experiments (seven in atherosclerosis group and eight in restenosis group). Finally, fifteen rats (eight in atherosclerosis group and seven in restenosis group) finished the experiment. Body weights and levels of blood glucose were shown in Table 1.

**TABLE 1:** The body weight and glucose levels in the diabetic rats at the start and end of experiment

Groups	Numbers	Initial weight (g)	Final weight (g)	Initial blood glucose (mmol/L)	Final blood glucose (mmol/L)
AS group	8	121.94 ± 22.04	420.1 ± 30.78	5.50 ± 0.61	23.78 ± 3.96
RS group	7	122.75 ± 32.29**	423.9 ± 33.07**	5.39 ± 0.45**	24.98 ± 4.17**

Abbreviations: AS group, atherosclerosis group; RS group, restenosis group.

** *P > *.05 vs AS group.

### 3.2 Colour doppler ultrasonogram

**FIGURE 1:**
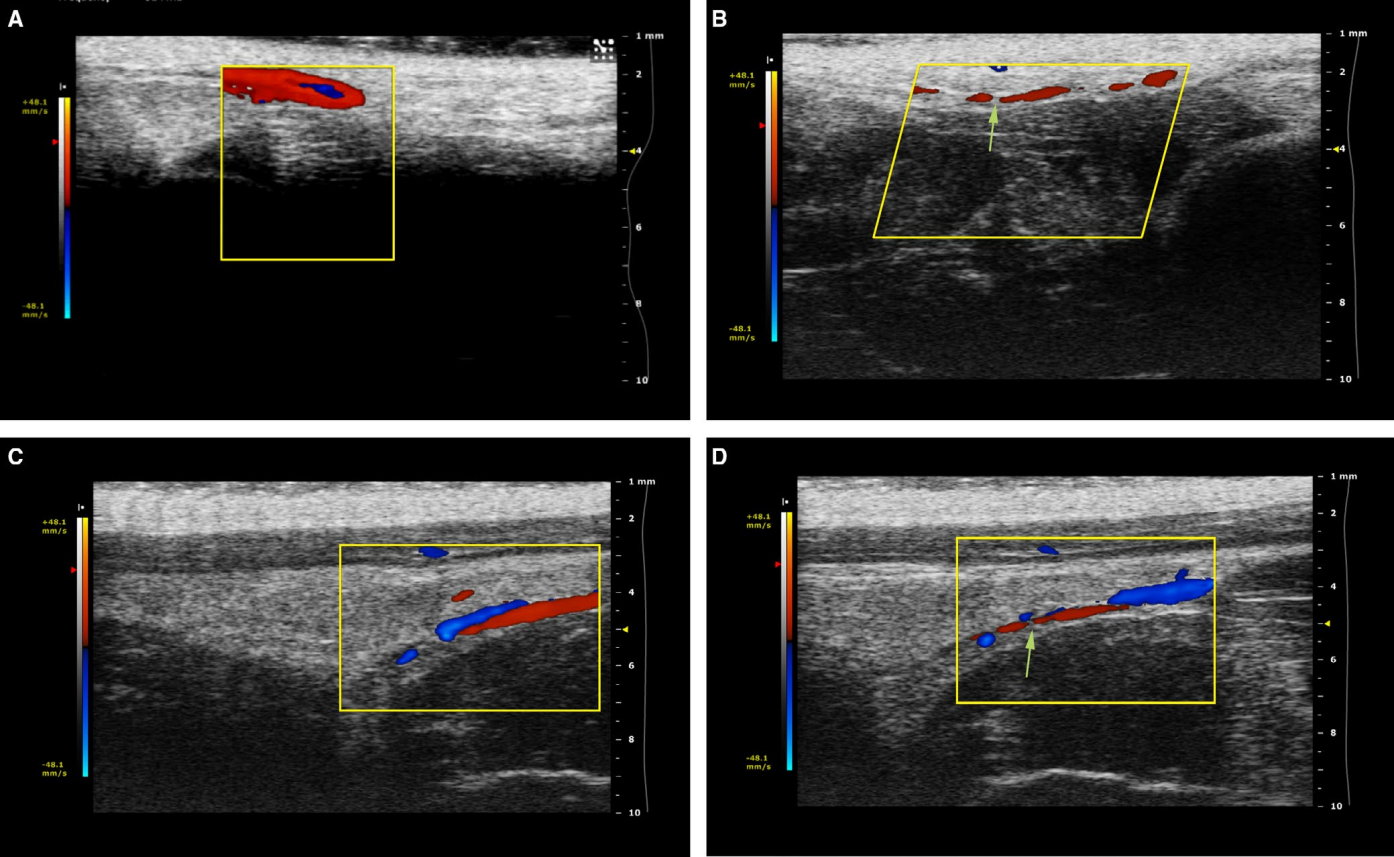
Results of colour Doppler ultrasonography demonstrated blood flow in different time points. A, Normal vessel, arterial blood (red) and venous blood (blue) flow; B, Four weeks after balloon‐induced endothelial injuries, and apparent discontinuation of arterial blood flow was observed (atherosclerosis damage was formed); C, Arteries recanalization of atherosclerosis plaque by ercutaneous transluminal angioplasty (PTA), and obstructed arterial blood vessel returned to recovery; D, Four weeks after PTA, iliac arterial blood flow blocked again and the restenosis plaque was established. When checking the arteries, the blood flow is directed towards the ultrasound probe. When checking the veins, the blood flow is facing away from the probe. Dashed lines indicated the position of the iliac artery. The yellow arrow indicated the formation of plaques and obstructed arterial blood flow, and scales were shown in the rightmost column of the image in red frame

Ultrasonography was to periodically assess the vascular status. After diabetes model was established, the iliac vessels of rats in restenosis group were damaged and subsequently observed with ultrasonography in different time points. Figure 1B showed that atherosclerosis plaques were established at four weeks after balloon injury surgery. Then the stenosed iliac arteries were recanalized after PTA surgery (Figure 1C), and restenosis plaques were formed at four weeks (Figure 1D).

### 3.3 VSMCs were significantly richer in restenosis plaques than atherosclerosis plaques

**FIGURE 2:**
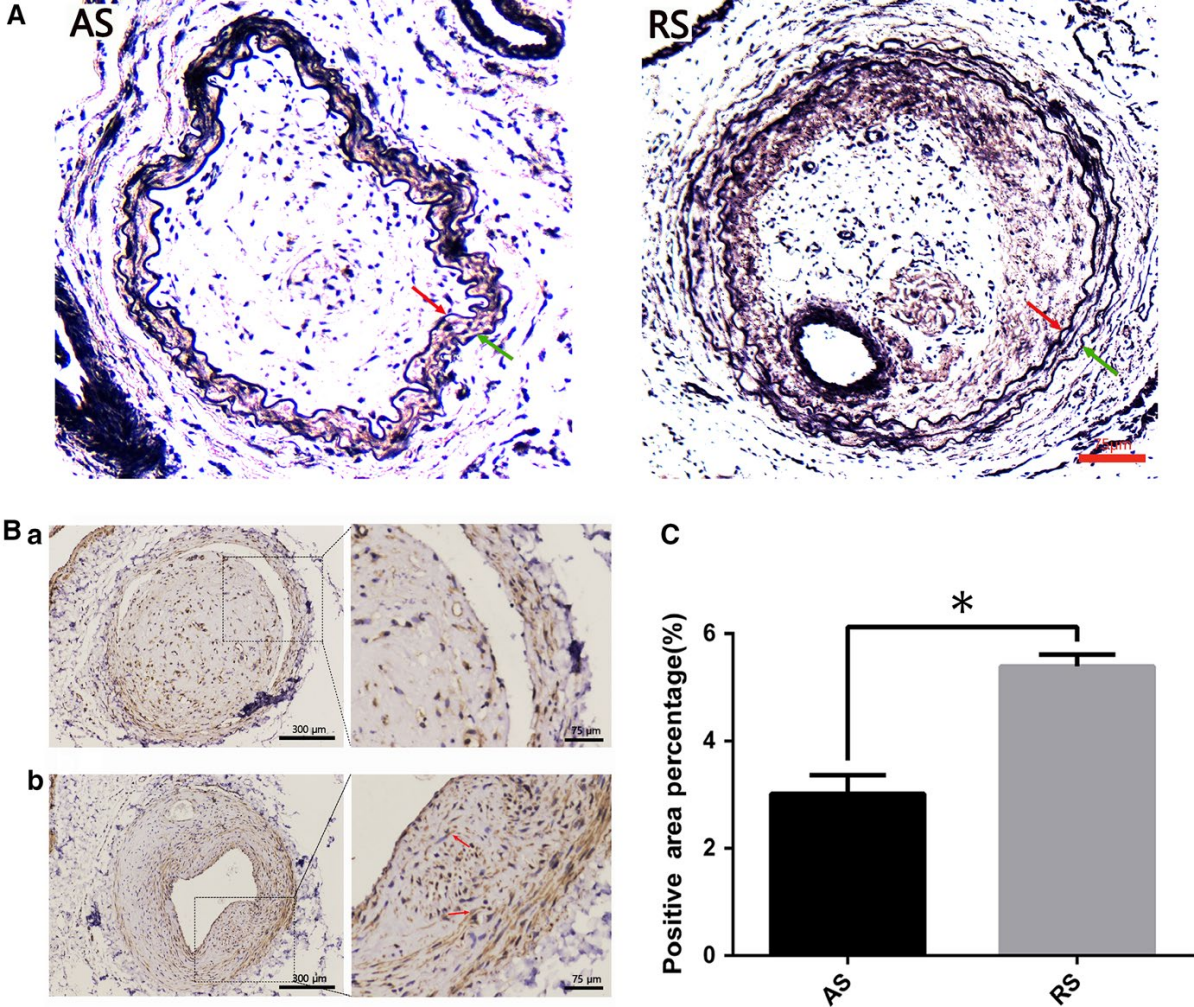
A was shown as overview of vessels. Immunohistochemistry staining for elastic‐van Gieson (EVG), EVG‐positive staining indicated the position of elastic tissue. The internal elastic lamina was labelled with red arrow and external elastic lamina was labelled with green arrow. AS, atherosclerosis group; RS, restenosis group. B, Immunohistochemistry staining for α‐SMA in atherosclerosis (a) and restenosis (b) plaques. α‐SMA was rarely observed in atherosclerosis plaque but filled in restenosis plaques. The red arrow indicated the positive staining for α‐SMA. The left images of immunohistochemistry staining for α‐SMA were taken at ×100 magnification and scale bar was 300 μm; the right part of picture A and B was magnified to ×200, respectively and scale bar was 75 μm. c, Quantification of positive staining for α‐SMA in atherosclerosis and restenosis groups. α‐SMA was significantly more in restenosis than atherosclerosis plaques. The values denote the positive area/ total area ± SEM. *Statistically significant difference (*P < *.05)

Samples were collected from the iliac arteries of rats in distinct groups. CD31 was used to exhibit the position of endothelial cells (Figure S1). The elastic membrane tissue was shown by elastic‐van Gieson (EVG) staining, and the intima is located in the inside of the internal elastic lamina. Figure 2A showed the apparent intimal hyperplasia both in atherosclerosis and restenosis groups. α‐SMA was the marker of VSMCs, and immunohistochemical analysis revealed that the expression of α‐SMA was significantly higher in restenosis plaques when compared to that of atherosclerosis plaques (*P < *.05, Figure 2B) by calculating the percentage through calculating the area of positive staining in terms of percentage of the total area.

### 3.4 Lin28a was differentially expressed in the plaques of restenosis and atherosclerosis

**FIGURE 3:**
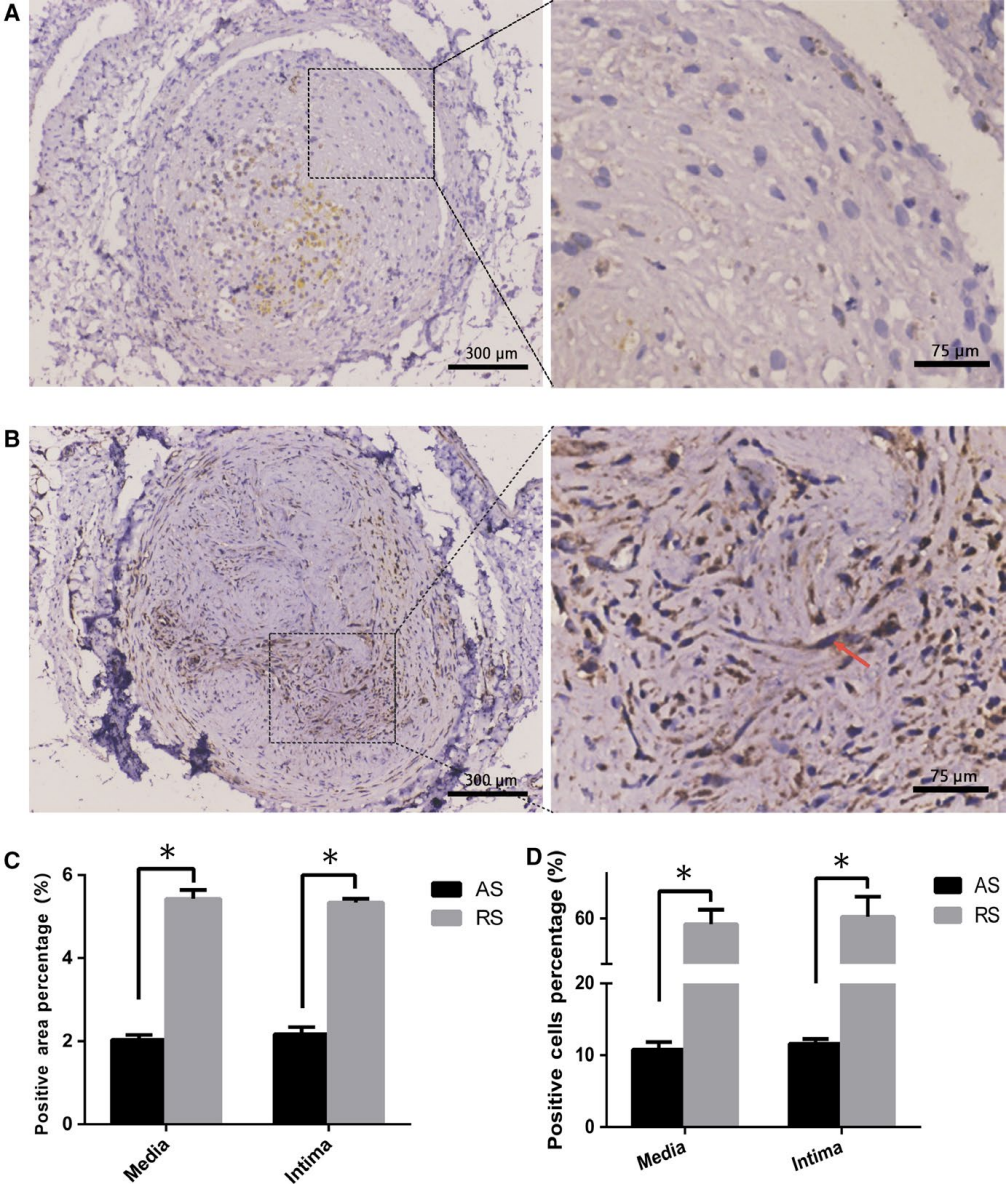
Immunohistochemistry staining for Lin28a in atherosclerosis (A) and restenosis (B) plaques. Few Lin28a was observed in atherosclerosis plaques but more Lin28a was filled with restenosis plaques. The red arrow indicated the positive staining for Lin28a. The left images of immunohistochemistry staining for Lin28a were taken at ×100 magnification and scale bar was 300 μm; the right part of picture A and B was magnified to ×200, respectively and scale bar was 75 μm. C and D, Quantification of positive staining area and cells for Lin28a in atherosclerosis and restenosis groups. The values denote the positive area/total area ± SEM. *Statistically significant difference (*P < *.05)

Since restenosis undergoes a process of tissue repair,^19^ Lin28a was demonstrated to enhances tissue repair.^14^ We then explored whether there existed differential expressions of Lin28a in restenosis and atherosclerosis plaques. Immunohistochemical results showed that Lin28a expression was significantly higher in restenosis plaques (Figure 3), while little was observed in atherosclerosis plaques by calculating the percentage of positive staining area in terms of total area. Additionally, Lin28a‐positive‐stained cells were significantly more in restenosis plaques (60.4 ± 2.6%) than that in atherosclerosis plaques (11.8 ± 3.1%), which further confirmed the results of positive staining area. Additionally, Lin28a was rarely expressed in the blank control group and diabetic control group (Figure S2).

**FIGURE 4:**
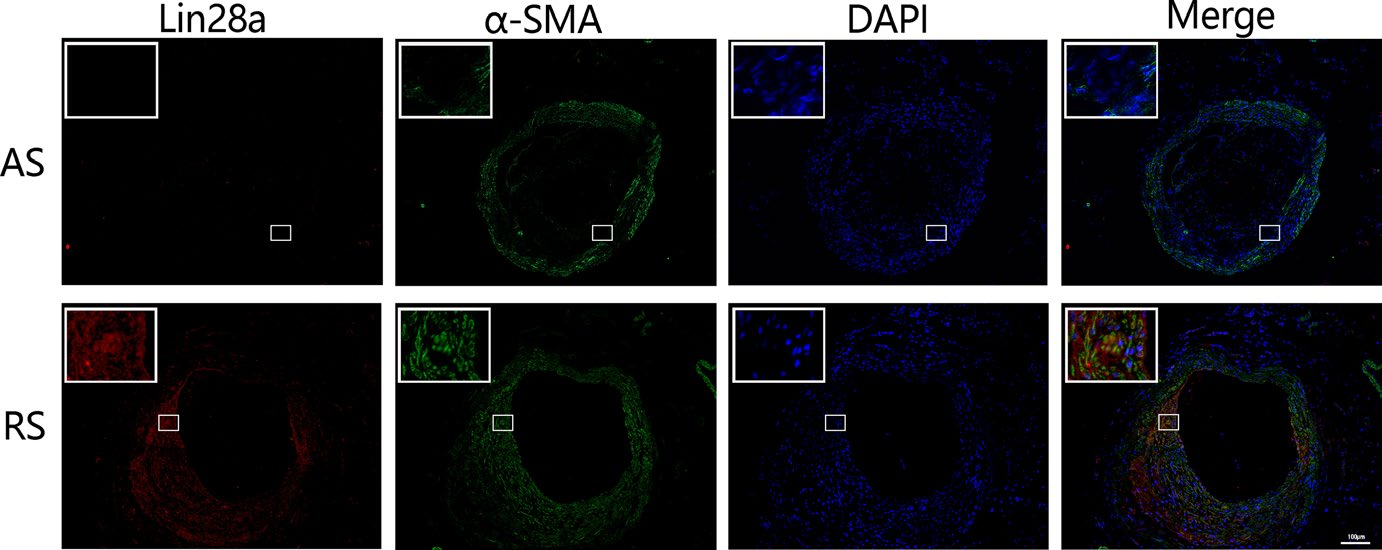
Expression and colocalization of Lin28a (red) and α‐SMA (green) in atherosclerosis (AS) and restenosis (RS) groups by immunofluorescence double staining. α‐SMA is the specific marker of vascular smooth muscle cells (VSMCs), and the merged orange colour represented the Lin28a expression in VSMCs. The merged orange colour was enhanced in RS group. The image of immunofluorescence was taken at ×100 magnification and scale bar was 100 μm; a small part was magnified to ×400 and is shown on the top left corner of each picture. AS, atherosclerosis group; RS, restenosis group

Furthermore, in order to verify the source of Lin28a‐positive cells in tissues, we performed immunofluorescence double staining for Lin28a and α‐SMA and found that Lin28a‐positive cells were stained as α‐SMA‐positive ones, which suggested that enhanced expression of Lin28a was closely related to the migration behaviour of VSMCs in restenosis plaques (Figure 4).

### 3.5 Lin28a was involved in the proliferation and migration of VSMCs

**FIGURE 5:**
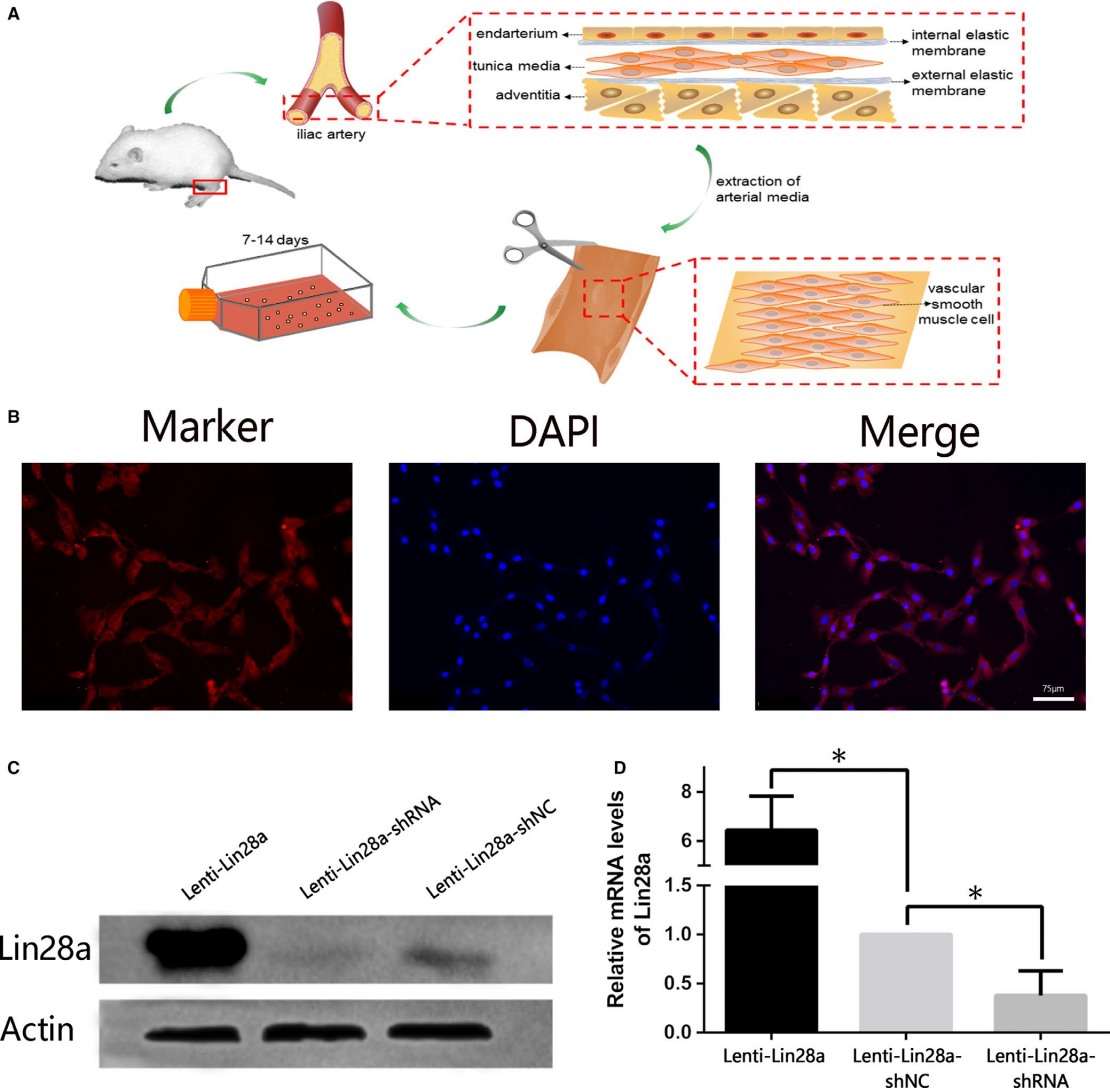
The isolation and verification of primary vascular smooth muscle cells (VSMCs) and regulation of Lin28a in VSMCs. A, The schema graph of primary VSMCs isolation. B, The verification of primary VSMCs by immunofluorescence stain for α‐SMA (red). The image of immunofluorescence was taken at ×200 magnification and scale bar was 75 μm. C, Lin28a protein expression was detected by western blot after regulated by Lenti‐Lin28a, Lenti‐Lin28a‐shRNA and Lenti‐Lin28a‐shNC, respectively. D, Lin28a mRNA expression was detected by qRT‐PCR after transfected by Lenti‐Lin28a, Lenti‐Lin28a‐shRNA and Lenti‐Lin28a‐shNC, respectively. *Statistically significant difference (*P < *.05)

**FIGURE 6:**
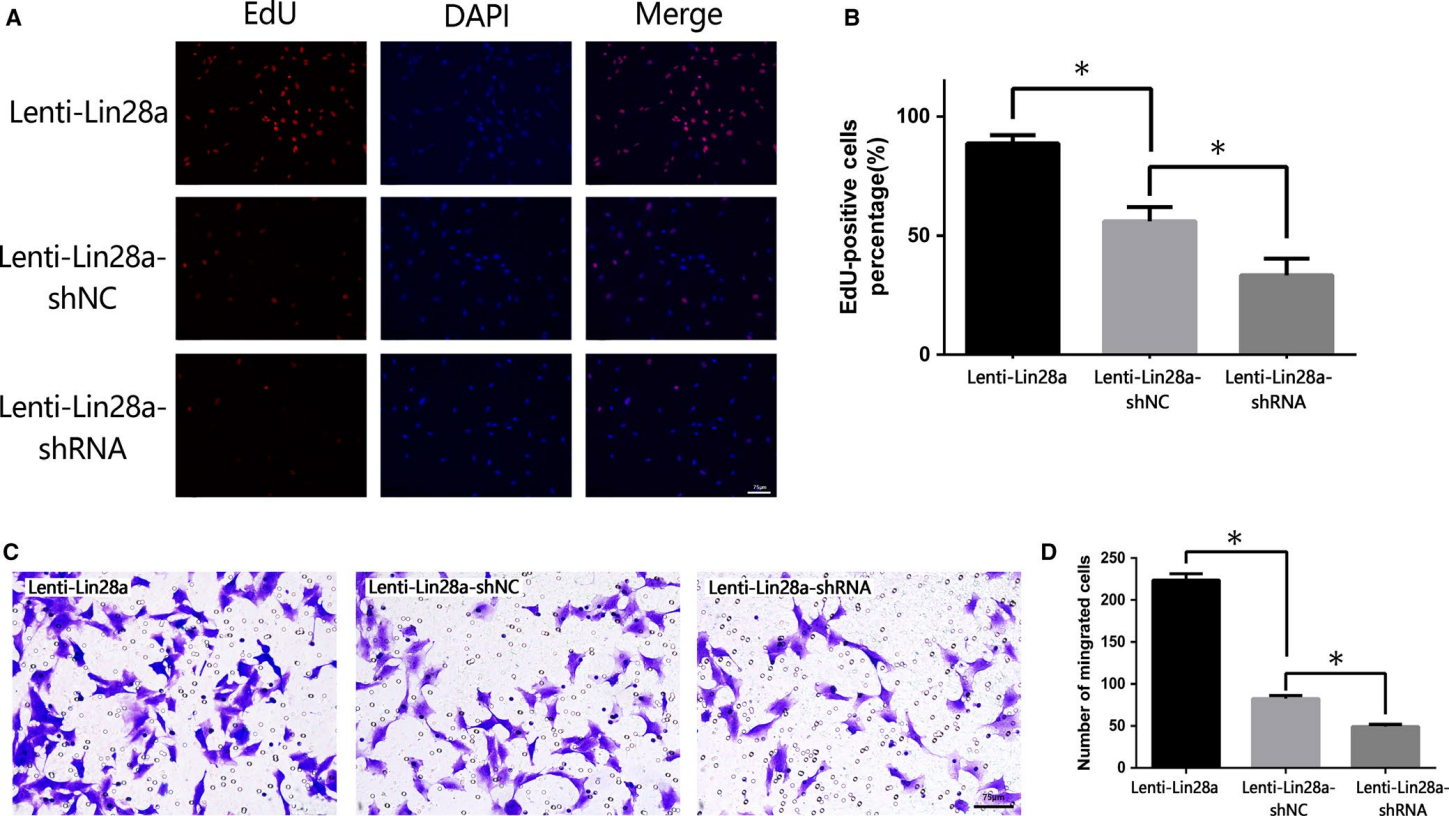
The proliferation and migration of VSMCs after regulation of Lin28a. A, EdU assay illustrated the proliferation of VSMCs. VSMCs were labelled with nucleoside analog EdU (red) for detection of DNA synthesis. Hoechst 33342 dye was used to visualize location of VSMCs nuclei (blue). The colocalization of EdU (red) and Hoechst 33342 (blue) was shown as pink colour, indicative of the proliferative VSMCs nuclei. B, Rate of EdU‐positive VSMCs. C, Transwell assay illustrated the migration of VSMCs. The migrated VSMCs were presented purple ones. D, The quantitative data of the migration of VSMCs. The images of EdU assay and Transwell migration assay were taken at ×200 magnification and scale bar was 75 μm. *Statistically significant difference (*P* < .05). VSMCs, vascular smooth muscle cells

In vitro, primary VSMCs which were extracted from iliac arteries of rats (Figure 5A) were verified by immunofluorescence staining for α‐SMA (Figure 5B) and SM22α (Figure S3). Subsequently, Lin28a expression in the primary VSMCs was examined after regulation. qRT‐PCR (*P < *.05, Figure 5D) and Western blot (*P < *.05, Figure 5C) results showed that Lin28a expression was effectively up‐regulated by transfected with Lenti‐Lin28a and down‐regulated by transfected with Lenti‐Lin28a‐shRNA, respectively. EdU assay showed that positive‐stained cells were obviously more after up‐regulation of Lin28a compared with the down‐regulation of Lin28a (Figure 6A). The result of Transwell migration assay in Figure 6C showed that the migrated cells labelled as the purple ones were significantly increased after overexpression of Lin28a. EdU and Transwell migration showed that the proliferation and migration of VSMCs were significantly increased with the up‐regulated Lin28a expression, and were significantly decreased with the down‐regulated expression of Lin28a (*P* < .05, Figure 6).

## 4 DISCUSSION

PTA is regarded as a vital treatment approach for patients with LEAD, especially for the patients with severe artery stenosis below popliteal artery.^20^ However, nearly half of the arteries after PTA develop restenosis in the first year following operation.^21^ To the best of our knowledge, there are few studies on the pathogenesis of restenosis after LEAD. Our previous studies found a unique mechanism of restenosis which was in consistence with others^22^, ^23^ and demonstrated that the excessive proliferation and migration of VSMCs from the media to the intima were the main cause of restenosis.^8^ In the present study, the potential pathogenesis was further investigated and we found that Lin28a played a crucial role in regulating the proliferation and migration of VSMCs in restenosis.

Lin28, a highly conserved RNA‐binding protein, was originally discovered in *C elegans* and it comprises a network of genes which can regulate the developmental timing.^11^ Studies showed that mutations of *Lin28* gene and other so‐called ‘heterochronic genes’ perturbed developmental progression in the worm, and these characteristic events occur either earlier or later than normal.^24^

Lin28 homologue involves Lin28a and Lin28b.^25^ A large amount of data focus on the pro‐neoplastic effect of Lin28b,^26^, ^27^ including colon tumours^26^ and oral squamous cell carcinoma.^27^ On the contrary, few studies focus on Lin28a. Lin28a is regarded as a principal element that participates in reprogramming mammalian somatic cells to pluripotent cells.^28^ Differ from Lin28b which is closely linked with malignant proliferation, Lin28a weights towards initiating and maintaining cell proliferation.^29^, ^30^ Hanna J et al previously indicated that the overexpression of Lin28a promoted cell division and led to the formation of induced pluripotent stem (iPS) cells more quickly.^31^ Furthermore, transgenic mouse that overexpressed Lin28a was reported to have enlarged brain size and body weight as well as slightly increased thickness of the cerebral cortex.^29^ Besides, the body size, crown‐rump length and growth rate of Lin28a transgenic mice were also augmented without malignant transformation.^32^ The above evidences presented the similarity to the process of restenosis plaques repair to some extent. Moreover, Lin28a expressed highly in the early stage of angiogenesis and it gradually decreased with the development of vascular maturation.^33^ Based on the above evidences, we hypothesize that Lin28a might be involved in restenosis formation.

Our previous study suggested that the plaque formation of restenosis and atherosclerosis involved different pathophysiological mechanisms. In the development of atherosclerosis, hyperglycaemia played a critical role, but had little effect on the formation of restenosis after PTA.^8^ Furthermore, we found that atorvastatin cannot arrest the occurrence of restenosis, which revealed blood lipids made rare difference on restenosis formation.^34^ For exploring the specific mechanism of restenosis formation, two distinct rat models were established. In our present research, abundant foam cells accumulation but few VSMCs appeared in the atherosclerosis plaques. However, VSMCs were proved to be the dominant components of restenosis plaques, and the excessive proliferation and migration of VSMCs from the media to the intima were found in restenosis plaques,^8^ which was in accordance with our present findings. Commonly, VSMCs mainly locate in the tunica media of the arteries, and are rarely observed in intima. Why do they migrate from tunica media to the intima after PTA? The underlying mechanisms involved in it were investigated and we found that expression of Lin28a was significantly increased in restenosis plaques, while it was hardly to find in atherosclerosis plaques.

Combined with the function in controlling angiogenesis,^33^ it is speculated that Lin28a played a key role in the formation and development of restenosis. As mentioned above, excessively migrated VSMCs are major elements of restenosis. Is Lin28a associated with VSMCs in restenosis? Our study showed that the Lin28a‐positive cells were also α‐SMA positive by double immunofluorescence staining, which suggested that enhanced Lin28a expression was closed related to the VSMCs in restenosis plaques. Subsequently, exact function of Lin28a on VSMCs was further examined in vitro. Our studies indicated that the proliferation and migration of VSMCs were markedly decreased by Lin28a down‐regulation. On the contrary, VSMCs showed a dramatic proliferation and migration trend with Lin28a up‐regulation, which was consistent with the increased pathological manifestations of VSMCs in restenosis plaques, and this further presented Lin28a an underlying key regulator in the restenosis disease.

The limitation of our study is that the signalling mechanism of Lin28a in restenosis was not performed, which need further investigations in our future study.

In summary, our studies highlighted the fact that Lin28a is critically involved in the progression of restenosis. VSMCs contribute significantly to restenosis plaques, which is different from atherosclerosis. In contrast to atherosclerosis, Lin28a was highly expressed in VSMCs of restenosis. In addition, the strongly positive correlation between Lin28a expression and proliferation and migration of VSMCs was found, which raised the possibility that Lin28a could be served as a novel therapeutic target for restenosis. A better understanding of the relationship between Lin28a and cell proliferation of VSMCs will make us have an in‐depth knowledge in the field of restenosis in LEAD.

## CONFLICT OF INTEREST

The authors confirm that there are no conflicts of interest.

## AUTHORS' CONTRIBUTIONS

In this work, Jianjun Dong and Lin Liao conceived the study and designed the experiments. Zhiwei Zou, Xiaojun Zhou, Qian Zhang and Rui Zhang performed the experiments and interpreted the results. Zhiwei Zou assisted in conducting the experiments and analysed the data. Xiaojun Zhou, Ruzhen Zhang, Shan Jiang and Chunmei Xu wrote the manuscript. Piyun Gong, Huangao Zhu, Dongmei Zhang, Tianyue Xie and Huimei Ma contributed to the critical revision of article. All authors read and approved the final manuscript.

## Supporting information

Figure S1

Figure S2

Figure S3
