# Supplementary figures and images for: Lin28a up‐regulation is associated with the formation of restenosis via promoting proliferation and migration of vascular smooth muscle cells
Source: J Cell Mol Med. 2020 Jul 25;24(17):9682–91. doi: 10.1111/jcmm.15506 (PMC7520293; doi:10.1111/jcmm.15506)

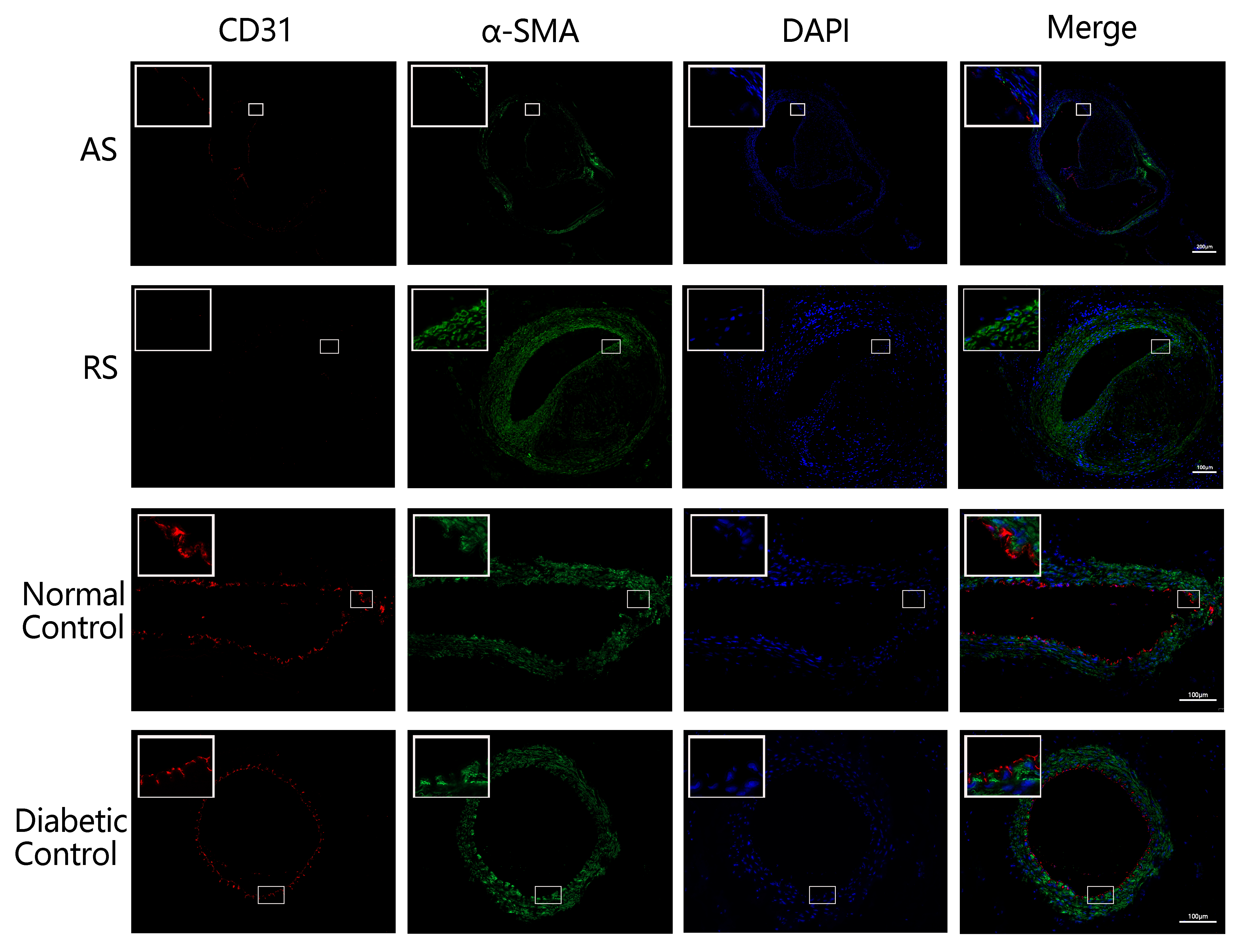

Supplement: Supplementary file 1 — Figure S1 [file JCMM-24-9682-s001.tif]

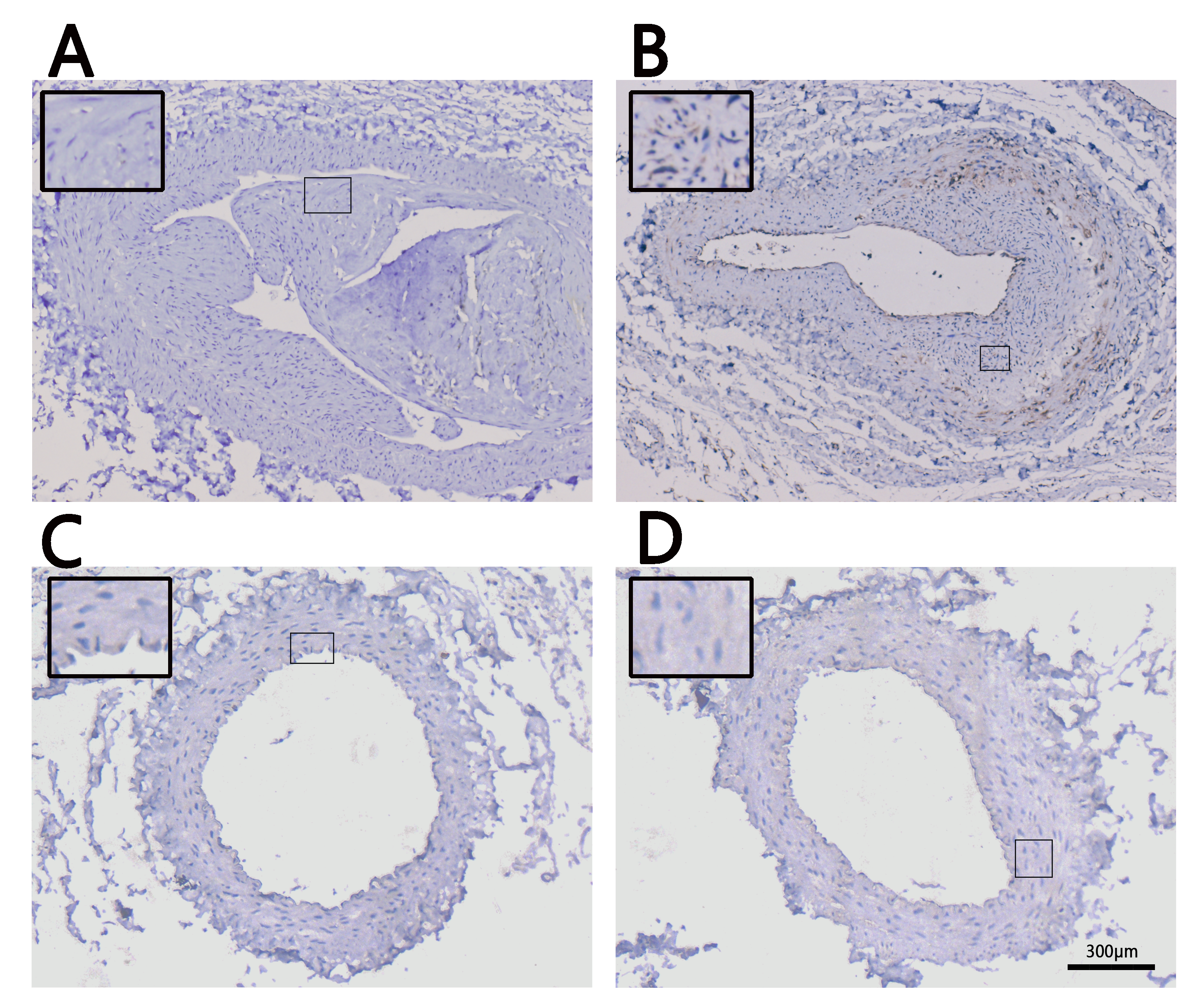

Supplement: Supplementary file 2 — Figure S2 [file JCMM-24-9682-s002.tif]

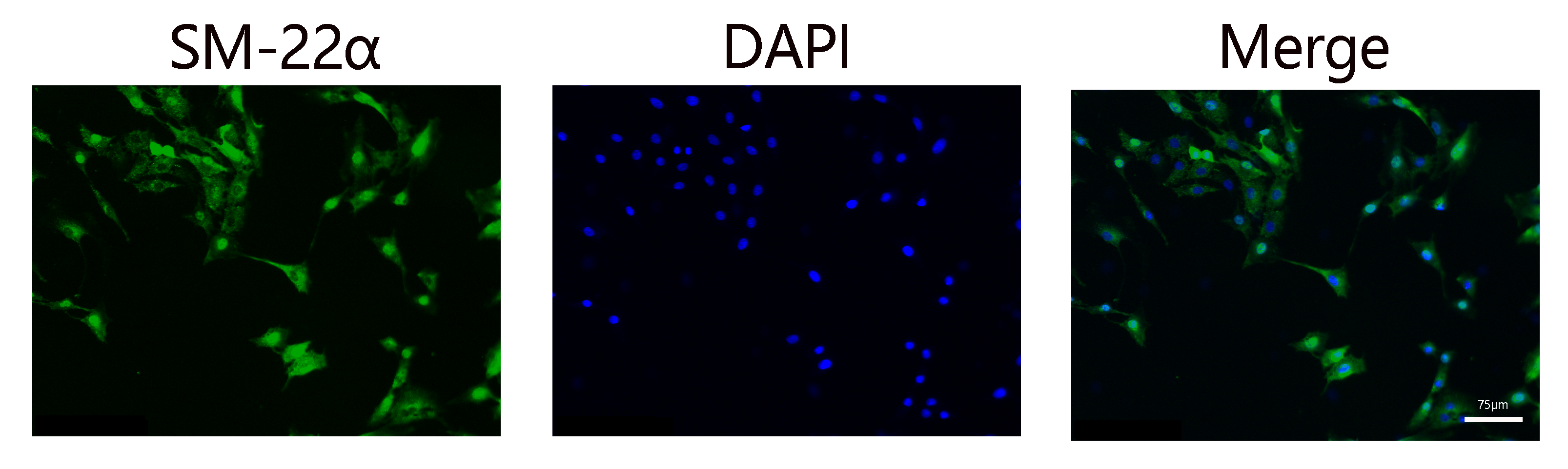

Supplement: Supplementary file 3 — Figure S3 [file JCMM-24-9682-s003.tif]
